# Olive Mill Wastewater-Loaded Polysaccharide Hydrogels as Potential Antibacterial Films for Wound Healing

**DOI:** 10.3390/gels12060549

**Published:** 2026-06-19

**Authors:** Eleonora Russo, Carla Villa, Anna Maria Schito, Debora Caviglia

**Affiliations:** 1 Department of Pharmacy, University of Genova, Viale Benedetto XV, 3, 16132 Genoa, Italy; carla.villa@unige.it (C.V.); debora.caviglia@edu.unige.it (D.C.); 2 Department of Integrated Surgical and Diagnostic Sciences, University of Genova, Viale Benedetto XV, 6, 16132 Genoa, Italy; anna.maria.schito@unige.it

**Keywords:** polysaccharide hydrogels, olive mill wastewater, wound healing, antibacterial films, bioadhesive materials, phenolic compounds, sustainable biomaterials

## Abstract

Polysaccharide-based hydrogels represent promising platforms for the development of bioactive wound dressings due to their biocompatibility, bioadhesive properties, and ability to maintain a moist environment at the wound interface. In this study, polymeric films were developed from natural polysaccharides incorporating olive mill wastewater (OMW) as a natural antibacterial agent. Chitosan (medium molecular weight), sodium alginate, sodium hyaluronate, and xanthan gum were selected to prepare hydrogel formulations either as single polymers or binary mixtures. Hydrogels were prepared by aqueous dispersion under magnetic stirring and subsequently converted into films using a solvent casting method. The resulting films were characterized in terms of rheological behavior, pH, morphology, thickness and water content. The obtained hydrogel films showed good casting ability, producing smooth and homogeneous matrices with adequate deformability and skin adhesion. Furthermore, they demonstrated a suitable capacity to absorb and retain water, mimicking the management of wound exudate. OMW was incorporated into the hydrogel formulations as a source of phenolic compounds with well-known antioxidant and antimicrobial properties. The presence of these bioactive compounds provides the films with potential antibacterial and antibiofilm activity against clinically relevant multidrug-resistant staphylococcal strains. These findings suggest that OMW-loaded polysaccharide hydrogels represent a promising and sustainable strategy for the development of antibacterial films for wound healing applications.

## 1. Introduction

Wound healing is a complex biological process involving a sequence of overlapping phases including hemostasis, inflammation, proliferation, and tissue remodeling [1,2]. The development of effective wound dressings plays a crucial role in promoting tissue repair and preventing infections, which remain among the main complications in skin injuries [3,4]. An ideal wound dressing should be non-toxic, non-allergenic, and non-adherent to the wound surface, while maintaining a moist environment that facilitates cell migration and tissue regeneration. Furthermore, it should be capable of absorbing wound exudates and should be easily removable without causing additional trauma to the damaged tissue [5,6].

In recent years, owing to their highly hydrated structure, hydrogels are increasingly investigated as wound dressing materials due to their high water content, biocompatibility, and ability to mimic the physiological environment of biological tissues [7,8]. Their crosslinked structure promotes water retention while preserving mechanical integrity, creating a moist microenvironment that promotes cell proliferation, migration, and tissue regeneration. Moreover, hydrogel-based dressings can absorb wound exudates, provide a physical barrier against microbial contamination, and allow gas exchange, essential factors for an efficient wound healing process [9,10,11].

Among the various hydrogel-forming materials, natural polysaccharides have emerged as particularly promising biomaterials for biomedical applications [12,13]. Polysaccharides represent an essential source of versatile natural polymers and are often considered superior to many synthetic materials due to their favorable physicochemical and biological properties [14,15]. These materials exhibit several advantageous characteristics, including excellent biocompatibility, biodegradability, low toxicity, and intrinsic bioactivity. In addition, many polysaccharides show good bioadhesive properties, enabling them to adhere to biological tissues and remain in close contact with the wound site, thereby improving the therapeutic effectiveness of the dressing [16,17].

Natural polysaccharides have been widely employed in wound care formulations owing to their ability to form hydrogels, thin films, and bioadhesive matrices capable of adhering to the wound surface and supporting the healing process [18,19]. Their structural versatility and functional groups allow the formation of flexible and biocompatible matrices capable of maintaining a moist environment at the wound interface while promoting tissue regeneration. Furthermore, some polysaccharides exhibit intrinsic antimicrobial activity, as reported for chitosan [20], which has been widely studied for its ability to inhibit microbial growth and enhance wound healing processes.

Polysaccharides used in biomedical and wound dressing applications can originate from a wide variety of natural sources, including marine algae, plants, animals, fungi, and bacteria. Among them, chitosan [21,22,23,24,25] sodium alginate [26,27,28,29], hyaluronic acid [30,31,32] and xanthan gum [33,34,35] are the most investigated polymers due to their favorable technological and biological properties. Chitosan (CHI) has been widely investigated in wound healing applications owing to its intrinsic antimicrobial activity, hemostatic effect, and capacity to support cellular proliferation and tissue repair. Alginate (ALGI), a polysaccharide derived from brown algae, is widely used in wound dressings due to its high capacity to absorb wound exudates and maintain a moist environment. Hyaluronic acid (HA) plays an important role in tissue repair and extracellular matrix organization, contributing to cell migration and angiogenesis during the healing process. Xanthan gum (GX), a microbial polysaccharide, has also attracted attention due to its excellent rheological properties, stability, and ability to form bioadhesive hydrogel systems. For these reasons, polysaccharide-based hydrogels represent an attractive platform for the development of innovative wound dressing systems. The combination of biocompatibility, bioadhesion, and structural versatility makes them suitable carriers for bioactive compounds, enabling the development of multifunctional materials capable of simultaneously protecting the wound, promoting tissue regeneration, and delivering therapeutic agents [36,37].

Combining bioactive substances of natural origin with polysaccharide hydrogel networks represents an effective strategy for designing advanced wound dressings capable of simultaneously providing structural support and promoting biological responses involved in tissue repair [38,39,40]. Among the natural sources of bioactive molecules, olive mill wastewater (OMW), a by-product of the olive oil production process, has recently attracted increasing scientific interest due to its high content of phenolic compounds. These molecules are well known for their antioxidant, anti-inflammatory, and antimicrobial properties, which may be particularly beneficial in the management of infected or chronic wounds. In particular, phenolic compounds derived from OMW have demonstrated promising biological activities against several human pathogens, including multidrug-resistant microorganisms [41,42].

Wound infections represent one of the main complications that can significantly delay the healing process and lead to severe clinical outcomes. The increasing emergence of antibiotic-resistant microorganisms has further complicated the management of infected wounds. Among these pathogens, multidrug-resistant staphylococcal strains are responsible for a large proportion of hospital acquired infections and are characterized by remarkable resistance to conventional antibiotic therapies. For this reason, the development of alternative antimicrobial strategies based on natural compounds has become an important area of research [43,44,45,46].

In this context, the incorporation of bioactive plants derived compounds into polymeric matrices represents a promising strategy for the development of multifunctional wound dressings. The combination of polysaccharide-based hydrogels with phenolic rich OMW could provide both structural and biological advantages, allowing the production of bioadhesive films with potential antibacterial activity [47,48].

Therefore, the aim of this study was to develop and characterize polysaccharide-based hydrogel films containing olive mill wastewater as a natural antibacterial component for wound healing applications. Hydrogels based on chitosan, sodium alginate, sodium hyaluronate, and xanthan gum were prepared and converted into films using a solvent casting method. The resulting hydrogels and films were evaluated in terms of physicochemical properties and suitability as wound dressing systems.

## 2. Results and Discussion

### 2.1. Rheological Behavior of Hydrogel Formulations

**Figure 1:**
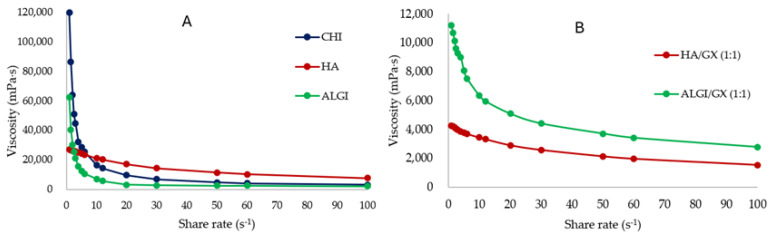
Flow curves of the hydrogel formulations used for film preparation. (**A**) Single-polymer hydrogels: CHI, chitosan; HA, sodium hyaluronate; ALGI, sodium alginate. (**B**) Binary hydrogels containing xanthan gum (GX): HA/GX (1:1) and ALGI/GX (1:1).

The rheological behavior of the hydrogel formulations used for film preparation was evaluated by measuring viscosity as a function of shear rate. As shown in Figure 1, all the formulations exhibited a decrease in viscosity with increasing shear rate, indicating a typical shear-thinning (pseudoplastic) behavior, which is characteristic of many polysaccharide-based hydrogels and is generally attributed to the progressive alignment and disentanglement of polymer chains under shear stress.

At low shear rates, the polymeric chains form a structured network stabilized by intermolecular interactions such as hydrogen bonding and chain entanglement, which results in higher viscosity values. As the shear rate increases, these interactions are progressively disrupted and the polymer chains tend to orient in the direction of flow, leading to a reduction in viscosity.

Among the tested formulations, the hydrogel-based on sodium hyaluronate (HA) showed relatively high viscosity values across the investigated shear rate range, indicating the presence of a well-structured polymer network. The sodium alginate (ALGI) hydrogel also exhibited a shear-thinning behavior, with a pronounced decrease in viscosity as the shear rate increased.

The addition of xanthan gum (GX) significantly affected the rheological properties of the hydrogels. In particular, the ALGI/GX (1:1) mixture showed the highest viscosity at low shear rates, indicating strong intermolecular interactions between the two polysaccharides. However, the viscosity decreased markedly with increasing shear rate, confirming the pseudoplastic nature of the system. Similarly, the HA/GX (1:1) formulation showed a moderate reduction with increasing shear rate, maintaining stable rheological properties suitable for film formation.

The formulations containing xanthan gum at a lower ratio (1:0.5) were not selected for subsequent characterization, as preliminary rheological evaluation and solvent casting experiments indicated lower film homogeneity and less favorable technological properties compared with the corresponding 1:1 system.

In contrast, the chitosan (CHI) hydrogel displayed lower viscosity values compared with the other formulations but maintained consistent shear-thinning behavior.

Based on the rheological results and on the film-forming ability observed during the solvent casting process, the three single-polymer hydrogels (CHI, ALGI, and HA) and the two binary mixtures (ALGI/GX 1:1 and HA/GX 1:1) were selected for film preparation. These formulations showed the most suitable viscosity profiles for the casting process and produced films with homogeneous surfaces, good moldability, and adequate adaptability, properties that are particularly desirable for wound dressing applications.

### 2.2. pH of Hydrogel Formulations

The pH values of the hydrogel formulations selected for film preparation ranged from 4.80 ± 0.02 to 7.05 ± 0.02, indicating good compatibility with the physiological pH range of the skin (Table 1). It should be noted that pH measurements were performed on the hydrogel formulations prior to film formation. Since the films were obtained after solvent evaporation, direct pH determination of the dry matrices was not feasible. Therefore, the pH values of the precursor hydrogels were used as an indicator of the suitability of the formulations for topical application and skin compatibility.

In particular, the CHI hydrogel showed the lowest pH value (4.80 ± 0.02). This slightly acidic pH may be advantageous for wound healing applications, as acidic environments are known to inhibit the growth of several pathogenic microorganisms and may contribute to restoring the natural acid mantle of the skin.

The HA and ALGI hydrogels exhibited nearly neutral pH values (7.05 ± 0.02 and 7.02 ± 0.01, respectively). Interestingly, the addition of xanthan gum in the binary mixtures resulted in a reduction in pH values, with HA/GX (1:1) and ALGI/GX (1:1) showing values of 6.13 ± 0.02 and 6.19 ± 0.01, respectively, indicating their suitability for topical application without causing significant irritation. Moreover, the incorporation of 10% (*w*/*w*) OMW into the hydrogel formulations did not significantly affect the pH values, indicating that the addition of OMW did not alter the acid–base balance of the systems. This result is particularly relevant from a formulation perspective, as it suggests that OMW can be incorporated as a bioactive component without compromising the physicochemical suitability of the hydrogels for wound dressing applications.

### 2.3. Film Morphology and Thickness

**Figure 2:**
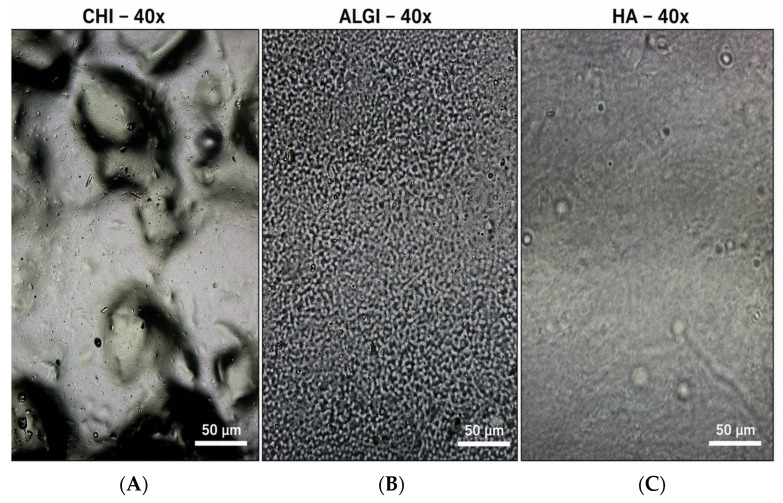
Optical microscopy images (40× magnification) of the solvent-cast films: (**A**) F-CHI, (**B**) F-ALGI, and (**C**) F-HA. Scale bar = 50 µm. The images reveal differences in surface morphology among the investigated formulations, with alginate- and hyaluronate-based films exhibiting more homogeneous and continuous surfaces compared with the chitosan-based film.

All the hydrogel formulations demonstrated good film-forming ability using the solvent casting method. Representative optical microscopy images of the films obtained are shown in Figure 2. The alginate- and hyaluronate-based films exhibited relatively homogeneous and continuous surfaces, with no evidence of major cracks or fractures. Small isolated microvoids were occasionally observed, most likely resulting from solvent evaporation during the drying process.

In contrast, the chitosan-based film displayed a less uniform morphology characterized by surface roughness and localized structural heterogeneities. Although no macroscopic defects were detected, the observed differences suggest that the nature of the polysaccharide significantly influences the organization of the polymeric matrix during film formation.

Film thickness shown in Table 2 ranged between approximately 0.010 and 0.020 mm depending on the polymer composition, indicating the formation of thin and uniform polymeric matrices suitable for topical applications.

Among the single-polymer systems, the ALGI-based film (F-ALGI) exhibited the highest thickness value (0.020 ± 0.005 mm), while the CHI (F-CHI) and HA (F-HA) films showed similar thickness values of approximately 0.015 mm.

The binary formulations containing xanthan gum (F-ALGI_GX_1_1 and F-HA_GX_1_1) resulted in slightly thinner films (0.010 ± 0.002 mm). This behavior may be attributed to the rheological properties of the mixed hydrogels, where the presence of xanthan gum improves the spreading ability of the polymeric solution during the solvent casting process, leading to the formation of more uniform and thinner films.

Overall, the obtained thickness values fall within the typical range reported for hydrogel-based films intended for wound dressing applications, where thin and flexible matrices are preferred to ensure good adaptability to the wound surface and improved patient comfort.

### 2.4. Film Water Loss

The water loss of the solvent-cast films ranged from 3.7 ± 0.6% to 8.2 ± 0.2%, depending on the hydrogel formulation and the drying conditions (Table 3).

In general, films stored in desiccators containing KOH showed higher weight loss values compared with those stored in CaCl_2_, indicating a stronger dehydrating effect of KOH under the experimental conditions.

Among the single-polymer films, F-ALGI exhibited the highest water loss (8.2 ± 0.2%) when stored with KOH, followed by F-CHI (8.0 ± 0.2%) and F-HA (7.5 ± 0.2%). This behavior may be related to the hydrophilic nature of these polysaccharides, which are able to retain and subsequently release significant amounts of water.

The presence of xanthan gum in the binary mixtures influenced the water loss behavior. In particular, F-ALGI_GX_1_1 showed the lowest water loss when stored with CaCl_2_ (3.7 ± 0.6%), suggesting that the interaction between alginate and xanthan gum may lead to the formation of a more compact polymeric network capable of retaining water more effectively.

**Figure 3:**
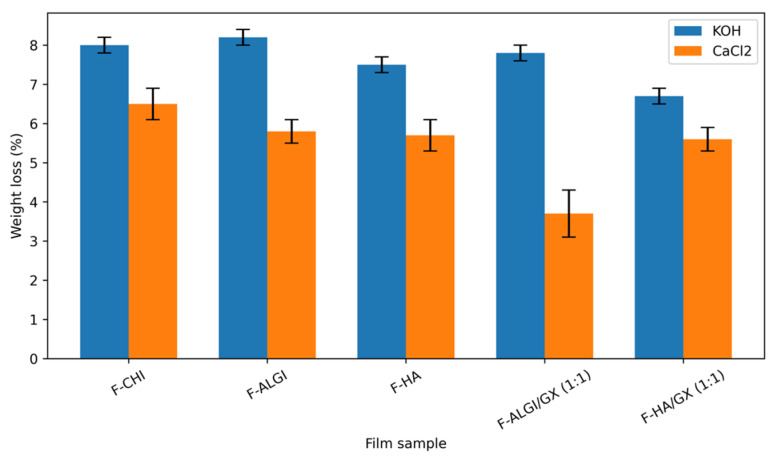
Water loss of solvent-cast hydrogel film under different drying conditions.

This trend is more clearly illustrated in the graphical representation of the data (Figure 3), where the differences between the drying conditions and the various film formulations can be easily compared.

Overall, the relatively low percentage of water loss observed for all formulations indicates that the obtained films maintain a suitable level of hydration, which is a desirable property for wound dressing applications, as hydrated polymeric matrices can promote better adhesion to the wound surface and support a moist healing environment.

Although differences in film thickness may partially contribute to the observed water loss values, the thickness range of the investigated films was relatively narrow (0.010–0.020 mm). Therefore, the observed differences are likely to be mainly associated with the intrinsic water-retention properties of the different polymeric matrices rather than with film thickness alone.

### 2.5. Water Uptake Capacity and Exudate Simulation

**Figure 4:**
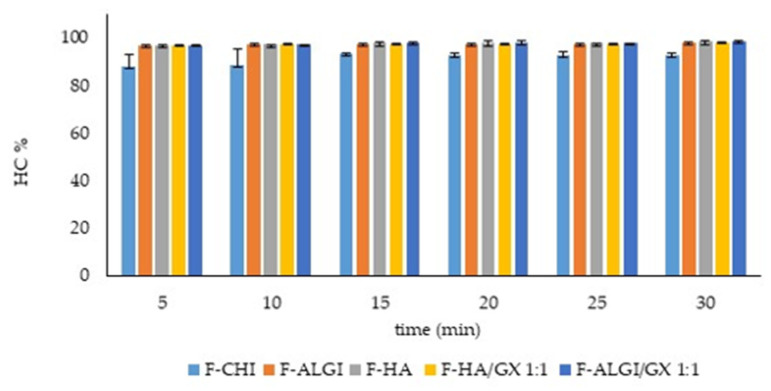
Hydration capacity (HC%) of the solvent-cast hydrogel films evaluated over time during exudate simulation studies. Data are expressed as mean ± standard deviation (SD) (n = 3). Error bars represent the standard deviation.

The hydration capacity (HC%) of the solvent-cast hydrogel films was evaluated over a time interval ranging from 5 to 30 min in order to simulate wound exudate absorption. As shown in Figure 4, all the investigated formulations exhibited high hydration capacity values throughout the entire experiment, confirming their strong affinity for aqueous media and their suitability for wound dressing applications.

Among the tested films, F-CHI showed the lowest HC% values at all time points, although the hydration capacity progressively increased over time, reaching values above 90% after 15 min. In contrast, the other formulations displayed higher hydration capacities after just 5 min of contact with water, with values generally ranging between approximately 95% and 99%.

The films containing alginate and hyaluronic acid, either alone or in combination with xanthan gum, exhibited rapid water uptake and maintained stable hydration values during the entire experimental period. In particular, the binary formulations F-HA/GX (1:1) and F-ALGI/GX (1:1) showed slightly higher HC% values compared to the corresponding single-polymer films, suggesting that xanthan gum contributes to the formation of highly hydrophilic and water-retaining polymeric networks.

Overall, the results demonstrate that all the developed films possess excellent water absorption and retention properties, which are essential characteristics for wound dressing materials intended to manage wound exudate while maintaining a moist environment favorable for tissue repair and regeneration. It should be noted that this assay was intended as a preliminary short-term evaluation of water uptake capacity. Further studies will be required to investigate film erosion, disintegration time, and matrix degradation over longer exposure times under simulated wound fluid conditions.

### 2.6. Antioxidant Activity of OMW-Loaded Hydrogels

**Figure 5:**
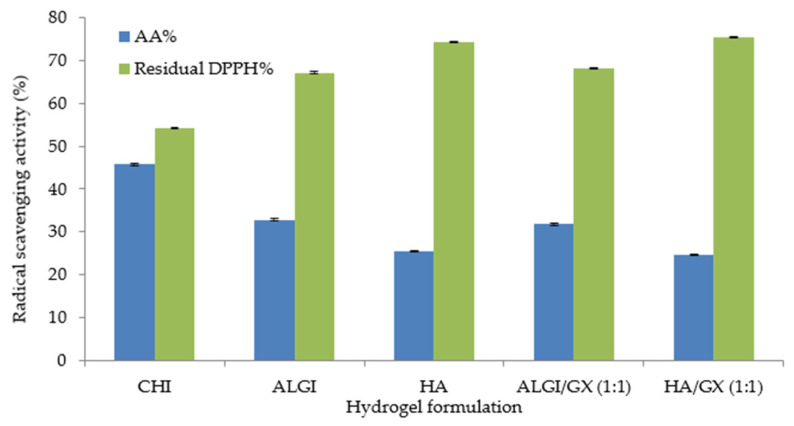
Antioxidant activity of the OMW-loaded hydrogel formulations evaluated by the DPPH assay. Blue bars represent the antioxidant activity (AA%), while gray bars indicate the residual DPPH percentage. Data are expressed as mean ± standard deviation (SD) (*n* = 3).

The antioxidant activity (AA%) of the 10% OMW-loaded hydrogel formulations was evaluated by the 2,2-diphenyl-1-picrylhydrazyl (DPPH) radical scavenging assay. As reported in Table 4 and in Figure 5, all the tested hydrogels exhibited moderate antioxidant activity, with AA% values ranging from approximately 24% to 46%.

Among the investigated formulations, the CHI hydrogel showed the highest antioxidant activity (45.76 ± 0.2%), indicating a more pronounced radical scavenging effect compared to the other systems. This result may be attributed to the combined contribution of the phenolic compounds present in OMW and the intrinsic properties of chitosan, which has been previously reported to exhibit antioxidant and bioactive characteristics.

The ALGI and ALGI/GX (1:1) formulations exhibited intermediate antioxidant activity values (32.85 ± 0.2% and 31.82 ± 0.2%, respectively), whereas the HA and HA/GX (1:1) hydrogels showed lower AA% values, ranging between 24% and 26%.

Overall, the obtained results demonstrate that the incorporation of OMW into polysaccharide-based hydrogels provides the formulations with significant antioxidant properties. The antioxidant activity values obtained in this study are comparable with those reported for polysaccharide-based hydrogels and films containing natural phenolic compounds and plant-derived antioxidants, confirming the ability of OMW to act as an effective source of radical scavenging molecules within the polymeric matrices [36,37,38,39,41].

This aspect is particularly relevant for wound healing applications, since antioxidant compounds may contribute to reducing oxidative stress at the wound site, thus promoting tissue regeneration and limiting inflammatory damage.

Statistical analysis revealed significant differences among the investigated formulations (one-way ANOVA, *p* < 0.0001). Tukey’s post hoc test showed that CHI exhibited significantly higher antioxidant activity than all the other formulations. No significant differences were observed between ALGI and ALGI/GX (1:1) or between HA and HA/GX (1:1), whereas ALGI-based systems showed significantly higher antioxidant activity than HA-based systems.

### 2.7. Inhibition of Biofilm Formation

The antibiofilm activity of the formulations loaded with OMW was evaluated against *Staphylococcus aureus* and *Staphylococcus epidermidis* strains using the crystal violet assay.

**Figure 6:**
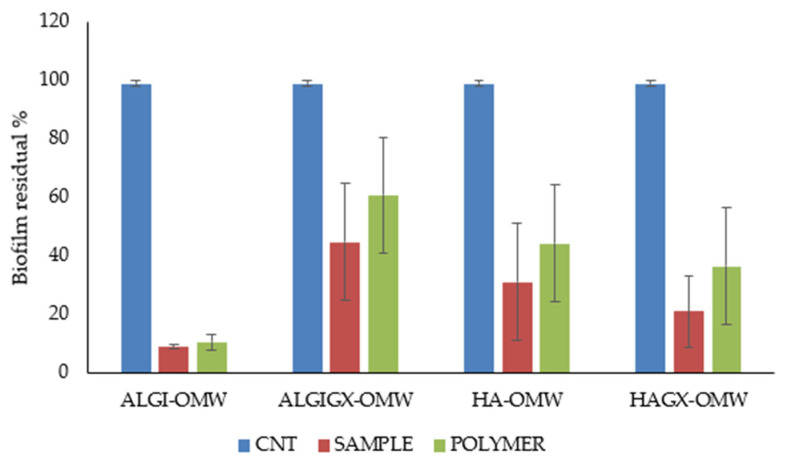
Antibiofilm activity of the tested formulations expressed as residual biofilm (%) relative to the untreated control (CNT). The reported values represent the mean biofilm inhibition obtained against the three bacterial strains tested in this study. Blue bars represent the control (CNT, 100% biofilm formation), red bars the hydrogel formulations (SAMPLE) containing olive mill wastewater (OMW), and green bars the corresponding hydrogel matrices without OMW (POLYMER). Data are reported as mean ± standard deviation (SD) (*n* = 3).

The results demonstrated that the incorporation of OMW significantly enhanced the ability of the polysaccharide-based systems to inhibit biofilm formation compared with the corresponding blank hydrogels. In Figure 6, the antibiofilm activity of the tested formulations, expressed as residual biofilm (%) relative to the untreated control (CNT), is reported. The reported values represent the average antibiofilm response obtained against the bacterial strains investigated in this study and were used to highlight the overall trend of biofilm inhibition induced by the different formulations.

Among the tested formulations, the ALGI-based hydrogels and the HA/GX formulations exhibited a marked reduction in biofilm biomass. The presence of xanthan gum may contribute to the stabilization of the polymeric network through intermolecular interactions with the other polysaccharides, thereby influencing the overall performance of the hydrogel system [33,35].

Formulations containing OMW (10%) exhibited higher antibiofilm activity compared to the corresponding systems without OMW, confirming the key role of phenolic compounds present in olive mill wastewater. These molecules are known to interfere with bacterial adhesion mechanisms and the early stages of biofilm formation.

Overall, all the tested samples showed a reduction in biofilm biomass compared to the untreated control, although with different inhibition profiles depending on the polymeric composition. In particular, the formulations containing OMW exhibited enhanced antibiofilm activity, suggesting that the phenolic compounds present in OMW contribute significantly to the inhibition of bacterial adhesion and biofilm formation.

The exclusion of chitosan-based systems from this assay highlights an important methodological limitation related to the interaction between chitosan and crystal violet, which may lead to an overestimation of biofilm biomass. Moreover, optical microscopy revealed a less homogeneous surface morphology for the chitosan-based film compared with the alginate- and hyaluronate-based systems.

Statistical analysis of the antibiofilm assay indicated an overall significant effect among the investigated formulations (one-way ANOVA, *p* < 0.05). However, Tukey’s post hoc test did not reveal statistically significant differences between individual polymeric systems, likely due to the variability among bacterial strains. Nevertheless, all OMW-loaded formulations showed a marked reduction in biofilm formation compared with the untreated control.

Overall, these findings indicate that OMW-loaded polysaccharide hydrogels are effective in preventing biofilm formation by clinically relevant staphylococcal strains. This behavior is particularly relevant for wound dressing applications, where biofilm formation is a major factor contributing to chronic infections and delayed healing.

## 3. Conclusions

The results obtained in this study demonstrate that polysaccharide-based hydrogels represent promising platforms for the development of multifunctional wound dressing films incorporating natural bioactive compounds. The formulations investigated exhibited suitable physicochemical and technological properties for topical applications, confirming the potential of natural polysaccharides as film-forming biomaterials.

Rheological evaluation revealed that all formulations exhibited a characteristic shear-thinning behavior, with viscosity decreasing as a function of increasing shear rate. Such a flow profile is advantageous for topical wound care systems, since it facilitates application and adaptation to the wound surface while preserving the cohesion and stability of the polymeric matrix. The pH values of the developed hydrogels were found to be within the physiological range of the skin, indicating good compatibility with topical administration. In particular, the slightly acidic pH observed for chitosan-based formulations may contribute to limiting bacterial proliferation, which is beneficial in the treatment of infected wounds.

The solvent casting technique proved to be a simple and effective approach for the preparation of homogeneous, flexible, and easily manageable films. In addition, the incorporation of xanthan gum improved the film-forming properties of the systems, probably due to enhanced intermolecular interactions and stabilization of the polymeric network. The developed films also exhibited excellent hydration capacity and water retention properties during exudate simulation studies. All formulations showed rapid water uptake and maintained high hydration values over time, indicating their ability to absorb and retain wound exudate while preserving a moist environment favorable for tissue repair. The incorporation OMW significantly enhanced the functional properties of the hydrogels. OMW is rich in phenolic compounds, including hydroxytyrosol and tyrosol, well known for their antioxidant and antimicrobial activities. The DPPH assay confirmed that these bioactive molecules retained their antioxidant capacity after incorporation into the polymeric matrices, with chitosan-based hydrogels showing the highest radical scavenging activity.

Furthermore, the antibiofilm results demonstrated that the presence of OMW markedly improved the antimicrobial performance of the formulations, suggesting a potential role of phenolic compounds in preventing bacterial colonization and biofilm formation at the wound site.

Overall, the combination of natural polysaccharide hydrogels containing OMW represents a promising and sustainable strategy for the development of innovative wound dressing systems with potential antioxidant and antibiofilm properties. Although the selected polysaccharides are widely recognized as biocompatible materials for topical applications, in vitro cytotoxicity assays on the final OMW-loaded films were not performed in the present preliminary study. Future investigations will include cytocompatibility studies on relevant skin cell lines, such as keratinocytes and fibroblasts, to further confirm the safety of the developed wound dressing systems.

## 4. Materials and Methods

### 4.1. Materials

Sodium alginate, sodium hyaluronate, and xanthan gum were purchased from Giusto Faravelli (Milan, Italy). Chitosan (medium molecular weight) was obtained from Sigma-Aldrich (St. Louis, MO, USA). Methanol, potassium hydroxide (KOH), calcium chloride (CaCl_2_), acetic acid, Trolox (6-hydroxy-2,5,7,8-tetramethylchroman-2-carboxylic acid), and DPPH (2,2-diphenyl-1-picrylhydrazyl) were supplied by Sigma-Aldrich. Deionized water was used for the preparation of all formulations.

The OMW used in this study was selected as a source of natural phenolic compounds and incorporated into the hydrogel formulations. According to previous investigations, olive mill wastewater is characterized by a total phenolic content typically ranging between 1.5 and 15 g GAE/L, depending on cultivar, geographical origin, and harvesting conditions. Since the biological activity of OMW is generally attributed to the synergistic action of its phenolic pool, no further quantification of individual phenolic compounds was performed in the present study.

### 4.2. Preparation of Hydrogels

Polysaccharide-based hydrogels were prepared by dispersing the selected polymer in the appropriate solvent under continuous magnetic stirring at room temperature. Hydrogels containing 0.5–2.5% (*w*/*w*) of polymer were obtained by dispersing sodium alginate, sodium hyaluronate, and xanthan gum in 100 mL of deionized water. Chitosan (2.5% *w*/*w*) was dissolved in 100 mL of aqueous solution containing 1 g of acetic acid (1% *w*/*v*) to ensure complete polymer solubilization. After complete dissolution of the polymer, OMW was incorporated into the hydrogels at a concentration of 10% (*w*/*w*) of the total formulation. OMW was added immediately after hydrogel preparation and homogenization. The resulting formulations (Table 5) were characterized shortly after preparation and subsequently used for film production by solvent casting. Hydrogels were stored at room temperature only for the time required for characterization and film preparation.

### 4.3. Preparation of Hydrogel Films

Hydrogel films were prepared using the solvent casting technique [49]. Previously prepared hydrogels based on chitosan (2.5% *w*/*w*), sodium alginate (2.5% *w*/*w*), sodium hyaluronate (2.5% *w*/*w*), and xanthan gum (0.5% *w*/*w*) were used as film-forming matrices.

In addition to the pure hydrogels (Table 5), binary mixtures were prepared only for sodium alginate (ALGI) and sodium hyaluronate (HA) by combining them with xanthan gum (GX) at polymer ratios of 1:1 and 1:0.5 (Table 6). Xanthan gum was used as a plasticizing agent to improve film flexibility and mechanical resistance.

Aliquots of hydrogel formulations were cast into circular Teflon molds (diameter 1.7 cm) and dried in an incubator at 37 ± 0.1 °C for 4–5 h. After drying, the films were removed from the molds and stored at room temperature in desiccators containing KOH or CaCl_2_.

### 4.4. Hydrogel and Film Characterization

#### 4.4.1. Rheological Analysis

Rheological measurements were carried out using a rotational viscometer (Viscostar-R, FUNGILAB S.A., Sant Feliu de Llobregat, Spain) equipped with a coaxial cylinder system (bob and cup geometry) and spindle R5. Approximately 22 g of each hydrogel were placed in Falcon tubes, and the flow behavior was evaluated by recording shear stress as a function of shear rate over a range of 1–100 s^−1^. All measurements were performed at 25 ± 0.5 °C in triplicate.

#### 4.4.2. pH Measurement

The pH of each formulation was measured at 25 ± 0.5 °C using a calibrated digital pH meter (Jenway 3510 Standard Digital pH Meter, Stone, UK). The electrode was directly immersed into the gel sample. Measurements were performed in triplicate, and results are expressed as mean ± standard deviation (SD).

#### 4.4.3. Film Morphology and Thickness

The morphology of the obtained films was evaluated by visual inspection and optical microscopy in order to identify possible defects such as cracks, fractures, pores, bubbles, or surface irregularities. Representative images were acquired using a Motic BA310E optical microscope (Motic, Barcelona, Spain) equipped with a digital imaging system, including a camera and display monitor, at 40× magnification. The films were carefully placed on glass slides and examined under reflected light to evaluate surface morphology, homogeneity, and the presence of structural defects.

Film thickness was measured using a digital Vernier caliper (TESA Technology, Renens, Switzerland). For each sample, the thickness was determined at the center of the film surface in order to ensure reproducibility of the measurement. The obtained values were expressed as mean ± standard deviation (SD) (*n* = 3).

#### 4.4.4. Film Water Loss

To determine the residual water content, each film was placed in a desiccator containing potassium hydroxide (KOH) or calcium chloride (CaCl_2_) as drying agents for 24 h. These desiccating agents were selected to evaluate the water loss behavior of the films under controlled low-humidity conditions and to assess their ability to retain moisture, an important parameter for wound dressing applications. The films were initially weighed (Wi) before being transferred to the desiccator and subsequently weighed again after the drying period (Wf).

All measurements were performed in triplicate (*n* = 3) and expressed as mean ± standard deviation (SD).

The weight loss percentage (WL%), corresponding to the residual water content, was calculated using the following Equation (1):

(1) $$\;\mathrm{WL}\;(\%)=\frac{W_{i}-W_{f}}{W_{i}}\cdot 100$$

where $W_{i}$ represents the initial weight of the film and $W_{f}$ the final weight after drying.

#### 4.4.5. Water Uptake Capacity and Exudate Simulation

The ability of the developed hydrogel films to absorb and retain aqueous fluids was evaluated in order to simulate wound exudate management. Pre-weighed dried films were placed in Petri dishes containing a fixed volume of distilled water and maintained at room temperature for different contact times (5, 10, 15, 20, 25, and 30 min).

At each predetermined time interval, the films were carefully removed, gently blotted with filter paper to eliminate excess surface liquid, and weighed again.

The hydration capacity percentage (HC%) was determined gravimetrically from the difference between the initial dry weight and the hydrated weight of the films according to the following Equation (2):

(2) $$HC(\%)=\frac{W_{h}-W_{d}}{W_{d}}\cdot 100$$

where $W_{h}$ is the hydrated film weight and $W_{d}$ is the initial dry film weight.

All measurements were carried out in triplicate and expressed as mean values ± standard deviation (SD).

### 4.5. Antioxidant Properties and Microbiological Evaluation

#### 4.5.1. Determination of Antioxidant Activity by the DPPH Assay

The antioxidant activity of OMW-loaded hydrogels was evaluated using the DPPH radical scavenging assay. Trolox solutions (ranging between 20 and 200 mg/L, R^2^ = 0.9995) were used to obtain a calibration curve. Samples, for the subsequent DPPH analysis, were prepared by diluting 0.6 g of hydrogel in a water/methanol mixture. Aliquots (0.1 mL) of each sample were mixed with 3.9 mL of DPPH solution and incubated in the dark for 30 min. Absorbance was measured at 516 nm using a UV–Vis spectrophotometer (Evolution 300, Fischer Scientific, GmbH, Schwerte, Germany). A control sample containing DPPH solution and solvent without hydrogel was used as reference.

The antioxidant activity (AA%) was calculated from the ratio of decreasing absorbance of sample solution (A_0_ − A_s_) to absorbance of blank DPPH solution (A_0_), as expressed in Equation (3) [50]:

(3) $$AA(\%)=\frac{A_{0}-A_{s}}{A_{0}}\cdot 100$$

All analyses were performed in triplicate (*n* = 3) and values are given ± standard deviation (SD).

#### 4.5.2. Evaluation of Biofilm Formation Inhibition

The antibiofilm activity of OMW-loaded hydrogel formulations was evaluated using a crystal violet (CV) staining assay in 24-well plate (Euroclone, Milan, Italy) method, based on crystal-violet staining, with spectrophotometric quantification of the adherent mass according to a procedure described by Cramton et al. [51].

Three bacterial strains were selected as representative models of clinically relevant Gram-positive biofilm-forming pathogens: one *Staphylococcus aureus* resistant to meticillin (MRSA, strain D) and two strains of *Staphylococcus epidermidis* (strains 2 and 22) both resistant to methicillin (MRSE). The three strains belonged to a collection of microorganisms from the DISC laboratories of the University of Genoa (Italy) and were isolated from human specimens and identified using the VITEK^®^ 2 technique (Biomerieux, Florence, Italy) or matrix-assisted laser desorption/ionization (MALDI-TOF) mass spectrometry (Biomerieux, Florence, Italy). Each bacterial suspensions were incubated overnight in Tryptic Soy Broth (TSB), supplemented with 0.25% glucose to promote biofilm production and subsequently diluted 1:100 in the same medium and added, in appropriate aliquots, in the wells of sterile 24-well plates.

Aliquots (300 µL) of each hydrogel formulation were deposited in triplicate in the wells and appropriate amounts of the diluted bacterial strains previously prepared were added. Control wells containing the bacterial strains considered were run in parallel. The tested formulations included sodium alginate (ALGI), sodium hyaluronate (HA), and their mixtures with xanthan gum (GX) (1:1 *w*/*w*), both in the absence and in the presence of olive mill wastewater. OMW was incorporated at a concentration of 10% (*w*/*w*) in all formulations [52].

Chitosan-based formulations were excluded from the antibiofilm assay due to their intrinsic interaction with crystal violet dye, which interfered with optical density (OD) measurements. After incubation at 37 °C for 24 h to allow biofilm formation, the wells were gently washed to washed three times with saline remove non-adherent cells. The remaining biofilm was stained with 0.1% (*w*/*v*) crystal violet for 30 min at room temperature. Plates were then washed with saline to remove excess of dye and then allowed to air dry. Solubilization of the bound dye permeated into the biofilm was obtained with 95% ethanol. The optical density (OD) of each well was measured spectrophotometrically at 570 nm by microplate spectrophotometer (CLARIOstar Plus—BMG LABECH, Ortenberg, Germany), and all readings were subtracted from the blank value, corresponding to the absorbance of wells containing only TSB-glucose treated with crystal-violet and ethanol. Each experiment was repeated at least three times. Biofilm formation was quantified by spectrophotometric measurement of crystal violet staining, providing a quantitative assessment of adherent biomass. All experiments were performed in triplicate to ensure reproducibility of the results.

The percentage of biofilm growth inhibition was calculated by the formula:

(4) $$BGI(\%)=\left[1-\left(At-Ac\right)\right]\cdot 100$$

where Ac is the average absorbance measured for the control wells and At is the average absorbance measured in the presence of the compound under study. 

### 4.6. Statistical Analysis

All results were reported as mean ± standard deviation (SD). Statistical analysis was performed using one-way analysis of variance (ANOVA) followed by Tukey’s multiple comparison test. Differences were considered statistically significant at *p* < 0.05. Analyses were performed using GraphPad Prism (version 8, GraphPad Software, San Diego, CA, USA).

## Figures and Tables

**Table 1:** pH values of the hydrogel formulations used for film preparation. Data are expressed as mean ± standard deviation (SD) (*n* = 3).

Hydrogel Formulations	pH
**CHI**	4.80 ± 0.02
**HA**	7.05 ± 0.02
**ALGI**	7.02 ± 0.01
**HA/GX 1:1**	6.13 ± 0.02
**ALGI/GX 1:1**	6.19 ± 0.01

**Table 2:** Thickness of the films obtained by solvent casting. Data are expressed as mean ± standard deviation (SD) (n = 3).

Film Solvent Casting	Thickness (mm ± SD)
**F-CHI**	0.015 ± 0.005
**F-ALGI**	0.020 ± 0.005
**F-HA**	0.015 ± 0.005
**F-ALGI_GX_1_1**	0.010 ± 0.002
**F-HA_GX_1_1**	0.010 ± 0.002

**Table 3:** Water loss (%) of the solvent-cast films after storage in desiccators containing KOH or CaCl_2_. Data are expressed as mean ± standard deviation (SD) (n = 3).

Film Sample	Drying Condition	Weight Loss (%)
**F-CHI**	KOH	8.0 ± 0.2
	CaCl_2_	6.5 ± 0.4
**F-ALGI**	KOH	8.2 ± 0.2
	CaCl_2_	5.8 ± 0.3
**F-HA**	KOH	7.5 ± 0.2
	CaCl_2_	5.7 ± 0.4
**F-ALGI_GX_ 1_1**	KOH	7.8 ± 0.2
	CaCl_2_	3.7 ± 0.6
**F-HA_GX_ 1_1**	KOH	6.7± 0.2
	CaCl_2_	5.6 ± 0.3

**Table 4:** Antioxidant activity of the OMW-loaded hydrogel formulations determined by the DPPH assay. Data are expressed as mean ± standard deviation (SD) (n = 3).

Hydrogel Formulation	Hydrogel Weight (g)	Absorbance (nm)	Residual DPPH (%)	Antioxidant Activity (AA%)
CHI	0.6	0.525	54.24 ± 0.2	45.76 ± 0.2
ALGI	0.6	0.650	67.15 ± 0.2	32.85 ± 0.2
HA	0.6	0.720	74.38 ± 0.1	25.62 ± 0.1
ALGI/GX (1:1)	0.6	0.660	68.18 ± 0.2	31.82 ± 0.2
HA/GX (1:1)	0.6	0.730	75.41 ± 0.1	24.59 ± 0.1

**Table 5:** Composition of the hydrogel formulations used in this study.

Blank Sample Code	Polymer Concentration	Solvent	Sample Code with 10% OMW
**CHI**	Chitosan 2.5%	Water/Acetic acid (1%)	**CHI/OMW**
**HA**	Sodium hyaluronate 2.5%	Water	**HA/OMW**
**ALGI**	Sodium alginate 2.5%	Water	**ALGI/OMW**
**GX**	Xanthan gum 0.5%	Water	–

**Table 6:** Composition of hydrogel formulations used for the preparation of solvent-cast films.

Film Code	Hydrogel Composition	Polymer Ratio (*w*/*w*)	Notes
**F-CHI**	Chitosan 2.5%	–	Single polymer
**F-ALGI**	Sodium alginate 2.5%	–	Single polymer
**F-HA**	Sodium hyaluronate 2.5%	–	Single polymer
**F-ALGI_GX_1_1**	Sodium alginate/Xanthan gum	1:1	Binary mixture
**F-ALGI_GX_1_0.5**	Sodium alginate/Xanthan gum	1:0.5	Binary mixture
**F-HA_GX_1_1**	Sodium hyaluronate/Xanthan gum	1:1	Binary mixture
**F-HA_GX_1_0.5**	Sodium hyaluronat/Xanthan gum	1:0.5	Binary mixture

## Data Availability

The original contributions presented in this study are included in the article. Further inquiries can be directed to the corresponding author.
